# The effectiveness of an add-on postbiotic to antipsychotic drugs for prevention of metabolic disturbances in patients with first-episode psychosis and schizophrenia spectrum disorder. A double blind clinical trial

**DOI:** 10.1192/j.eurpsy.2025.1680

**Published:** 2025-08-26

**Authors:** J. Chato Noriega, E. Giné Servén, A. Ballesteros, M. Araña, M. Barajas, D. Yavorov, I. Iturria, J. Ayo, J. García, G. Núñez, M. J. Cuesta, E. Rosado, A. M. Sánchez Torres, G. Gil, X. Ansorena, I. J. Encio

**Affiliations:** 1 Instituto de Investigación Sanitaria de Navarra (IdiSNA),; 2 Department of Psychiatry, Hospital Universitario de Navarra; 3 Universidad Pública de Navarra; 4 Genbioma Aplicaciones SL; 5 Intelligent System Vitale SL; 6 Instituto de Investigación Sanitaria de Navarra (IdiSNA), Pamplona, Spain

## Abstract

**Introduction:**

Atypical antipsychotics (APs) are the drugs of choice for the treatment of the acute episodes and for relapse prevention in schizophrenia (SZ) and psychosis. Nevertheless, these drugs have side effects and particularly increase the risk to develop metabolic syndrome. Moreover, it has been reported that first-episode psychosis (FEP) patients have significant trends to insulin resistance, a higher body mass index and a higher rate of obesity, compared to the healthy subjects. Changes in gut microbiome have been linked to increased systemic inflammation, which could be associated with metabolic disturbances and the development of SZ. In this context, some previous studies have explored the efficacy of probiotic supplementation in SZ, showing benefits in gut regulation and in improving the metabolic effects of APs.

**Objectives:**

The purpose of this study is to evaluate the effectiveness of an add-on postbiotic to Aps on metabolic disturbances and psychopathological variables in patients diagnosed with FEP or schizophrenia spectrum disorder (SSD). , as well as to determine whether the addition of postbiotics can improve biomarkers related to compensatory immunity and the endocannabinoid system.

**Methods:**

A randomized, double-blind, placebo-controlled clinical trial, in which postbiotic or placebo will be administered for 12 weeks as add-on APs. The study comprises two branches: FEP branch, patients recently diagnosed with first psychotic episode; and SSD branch, patients with long-standing psychotic disorder. Five follow-up appointments will be conducted along the 12 weeks to carry on clinical assessments. Patients will be monitoring with a glucose sensor, and blood and microbiota will be analysed.

**Results:**

This is a study protocol that is currently underway. No results are available at this time.

**Conclusions:**

Over the past few decades, it has been abundantly evident how important the human microbiota is to both short-term and long-term human health. In this regard, postbiotics seem to have higher benefitial effects and lower risk than probiotics and they offer a promising approach to improve metabolic disturbances and amelioration of psychopathological symptoms in FEP and SSD patients.

**Disclosure of Interest:**

None Declared

